# Hepatitis E Virus in Rats, Los Angeles, California, USA

**DOI:** 10.3201/eid1712.110482

**Published:** 2011-12

**Authors:** Robert H. Purcell, Ronald E. Engle, Michael P. Rood, Yamina Kabrane-Lazizi, Hanh T. Nguyen, Sugantha Govindarajan, Marisa St. Claire, Suzanne U. Emerson

**Affiliations:** National Institutes of Health, Bethesda Maryland, USA (R.H. Purcell, R.E. Engle, Y. Kabrane-Lazizi, H.T. Nguyen, S.U. Emerson);; Department of Public Health Services, Los Angeles, California, USA (M.P. Rood);; Rancho Los Amigos Hospital, Downey, California, USA (S. Govindarajan);; Bioqual, Inc., Rockville, Maryland, USA (M. St. Claire)

**Keywords:** zoonoses, serial passage, virus sequence, viruses, rats, hepatitis E virus, Los Angeles, California

## Abstract

The role of rats in human hepatitis E virus (HEV) infections remains controversial. A genetically distinct HEV was recently isolated from rats in Germany, and its genome was sequenced. We have isolated a genetically similar HEV from urban rats in Los Angeles, California, USA, and characterized its ability to infect laboratory rats and nonhuman primates. Two strains of HEV were isolated from serum samples of 134 wild rats that had a seroprevalence of antibodies against HEV of ≈80%. Virus was transmissible to seronegative Sprague-Dawley rats, but transmission was spotty and magnitude and duration of infection were not robust. Viremia was higher in nude rats. Serologic analysis and reverse transcription PCR were comparably sensitive in detecting infection. The sequence of the Los Angeles virus was virtually identical to that of isolates from Germany. Rat HEV was not transmissible to rhesus monkeys, suggesting that it is not a source of human infection.

## Abstract 2

This virus is unlikely to be a zoonotic threat.

Hepatitis E virus (HEV) is a major cause of epidemic waterborne and sporadic hepatitis in developing countries. Hepatitis E is caused principally by HEV genotypes 1 and 2 (*1*). Recently, hepatitis E has been diagnosed with increasing frequency as a cause of sporadic hepatitis in industrialized countries (*2*). Additionally, a large proportion (<20%) of populations of such countries have antibodies against HEV in the absence of any recognized hepatitis (*3**–**5*), and evidence is increasing that these antibodies might be the result of subclinical infections acquired zoonotically.

Strains of HEV representing genotypes 3 and 4, which have been isolated from humans with hepatitis E, regularly infect pigs worldwide (*6*), and infection in humans caused by eating undercooked meat from domestic pigs, wild boar, and several species of wild deer has been documented (*6**,**7*). However, many, if not most, persons who have unexplained antibodies against HEV do not eat undercooked pork or venison, raising the possibility that other animals or modes of zoonotic transmission exist. It is noteworthy that swine handlers in the United States have a higher incidence of antibodies against HEV than do healthy blood donors, even though pork is generally thoroughly cooked in the United States. Therefore, eating pork is unlikely to explain the prevalence of antibodies against HEV in this country.

Numerous species, including rodents, have been found to have antibodies reactive with capsid protein of human HEV strains, and HEV closely related to genotypes 3 or 4 has been recently isolated from rabbits (*8*), cattle (*9*), and sheep (*10*). However, an HEV strain recently isolated from rats was unique and only distantly related to known strains (*11*). Thus, it is important to understand how this rat virus is related to human infections. Rats are particularly interesting as a potential source of human infections because although they are not a human food, they have a high seroprevalence of antibodies against HEV (*12**,**13*) and they are ubiquitous and in close contact with humans everywhere.

We have demonstrated that a high proportion of wild-caught *Rattus norvegicus*, *R. rattus*, and *R*. *exulans* rats trapped in several US cities (Baltimore, Maryland; New Orleans, Louisiana; and the islands of Oahu and Hawaii, Hawaii) were positive for antibodies against HEV (*12*). We studied their seroepidemiology but were unable to obtain genomic sequence or to transmit an agent to laboratory rats. Subsequently, in collaborations with the County of Los Angeles Department of Health (Los Angeles, CA, USA) Vector Management Program, we succeeded in transmitting to laboratory rats 2 strains of HEV from rats from Los Angeles but were again unable to obtain genomic sequence (*14*).

Recent cloning of rat HEV obtained from *R. norvegicus* rats in Germany and development of more broadly reactive PCR primers (*11*) prompted us to revisit those experiments. This report describes the partial PCR amplification and characterization of a US strain of rat HEV.

## Materials and Methods

### Rat Serum

Wild *R. norvegicus* rats were live-trapped by vector-control personnel in urban Los Angeles. The rats were anesthetized, and age and species was determined. Reproductive status and weight were recorded. Blood was obtained by cardiac puncture, and the serum was stored at −70°C.

### HEV Strains

We performed transmission studies with genotype 1 strains Sar-55 (*15*), Kashi-87 (*16*), Akluj-90 (*17*); genotype 3 strain Meng swine HEV (*18*); and genotype 2 strain Mex 14 (*19*). All strains were in 10% fecal suspensions, diluted as described in the Results, and all but 1 had been titered for infectivity in nonhuman primates or pigs (Table 1).

**Table 1:** Results of testing for transmission of human and swine HEV to laboratory rats, Los Angeles, California, USA*

Inoculum	Genotype	ID_50_	No. injected	No. with HEV RNA or antibodies against HEV
Sar 55	1	10^3.8^†	2	0
Akluj-90	1	10^4.8^†	2	0
Kashi-87	1	10^8.1^‡	2	0
Mex14	2	10^4.3^†	4	0
Meng	3	10^4.3^§	4	0
Meng	3	10^2.3^¶	4	0

*HEV, hepatitis E virus. The 50% infectious dose (ID_50_) was administered intravenously. †In human feces and titered in macaques. ‡Quantitative reverse transcription PCR titer. §In pig feces and titered in pigs. ¶In macaque feces and titered in macaques.

### Transmission Studies

Because infectivity of HEV in nonhuman primates is ≈10,000-fold less when administered orally than when administered parenterally, commercially acquired, outbred, Sprague-Dawley (*R. norvegicus*) or athymic nude hooded laboratory rats (Harlan, Indianapolis, IN, USA) or rhesus monkeys (*Macaca mulatta*) that were bred and raised in captivity were infected intravenously with serum or homogenized 10% fecal or tissue samples in saline. The animals were housed and maintained at Bioqual, Inc. (Rockville, MD, USA). Housing and care of the animals complied with all relevant guidelines and requirements, and the animals were housed in facilities that are fully accredited by the Association for Assessment and Accreditation of Laboratory Animal Care International. All protocols were reviewed and approved by the Institutional Animal Care and Use Committees of the National Institute of Allergy and Infectious Diseases of the National Institutes of Health (Bethesda, MD, USA) and Bioqual, Inc.

Blood samples were obtained weekly and feces were obtained daily to 3×/wk. Serum samples were tested for liver enzyme levels by using standard methods (AniLytics, Inc., Gaithersburg, MD, USA). Postmortem liver tissue was fixed in formalin, embedded, sectioned, and stained with hematoxylin and eosin (American Histo Laboratories, Inc., Gaithersburg, MD, USA) and read under code by one of the authors (S.G.). Samples were scored for liver pathologic changes by the histologic activity index method.

### Serologic Tests

Serum samples were tested for IgG and IgM isotypes against HEV by using a peroxidase-based ELISA as reported (*12*). The antigen used was recombinant open reading frame 2 protein of genotype 1. Serum samples were tested at 10-fold dilutions, and the highest dilution exceeding the cutoff value of optical density was taken as the endpoint titer of the serum.

### Nested Reverse Transcription PCR

RNA was extracted from 270 µL of serum by using Trizol LS (Invitrogen, Carlsbad, CA, USA), and purified RNA was resuspended in 20 μL of water. Nested reverse transcription PCR (RT-PCR) was performed with the same primers, enzymes, and thermal profiles as described (*11*). Nested PCR products were separated by electrophoresis on ethidium bromide–stained agarose gels, extracted from the gel, and sequenced to provide the consensus sequence.

### Quantitative RT-PCR

RNA was extracted from 50 μL of serum, tissue suspension, or filtered (0.22 μm, UltrafreeMC; Millipore, Billerica, MA, USA) 10% fecal suspension by using the QIAamp Viral RNA Mini Kit, (QIAGEN, Valencia, CA, USA), and total RNA was eluted in a volume of 60 μL. A TaqMan assay was performed by using the 7900HT Real-Time PCR System (Applied Biosystems, Foster City, CA, USA) according to the manufacturer’s recommendations. The primers (for a 332-base amplicon) consisted of 900 nmol/L forward (5′-ATG GTG CTT TTA TGG CGA TTG-3′) and 900 nmol/L reverse (5′-CAA ACT CAC TGA AAT CAT TCT CAA AAA C-3′), and 250 nmol/L probe (5′-6FAM-TAT GTT CAG GAG AAG TTG GAA GCC GCT GT-TAMRA-3′). One-step quantitative RT-PCR (qRT-PCR) cycling conditions were 15 min at 48°C, a 10-min incubation at 95°C, and 50 cycles for 15 s at 95°C and 1 min at 60°C. Rat TaqMan cycle threshold values were indirectly quantified against an in-house HEV genotype 1 quantity standard line that represented a 6-log dynamic range.

## Results

### Detection of Rats Infected with Human- or Swine-derived HEV Strains

Because isolation of mammalian HEV strains from rats had been reported, we attempted to transmit to laboratory rats 6 well-characterized mammalian HEV strains (genotypes 1, 2, and 3) that can infect primates or pigs (Table 1). Adult Sprague-Dawley rats were injected intravenously with 0.1 mL of inoculum through the tail vein. Rats were bled weekly for 16 weeks and monitored for HEV RNA by real-time PCR with genotype-specific primers and for development of antibodies against HEV by ELISA. None of the animals had any evidence of infection.

### Isolation of HEV Strains from Wild Rats

**Figure 1:**
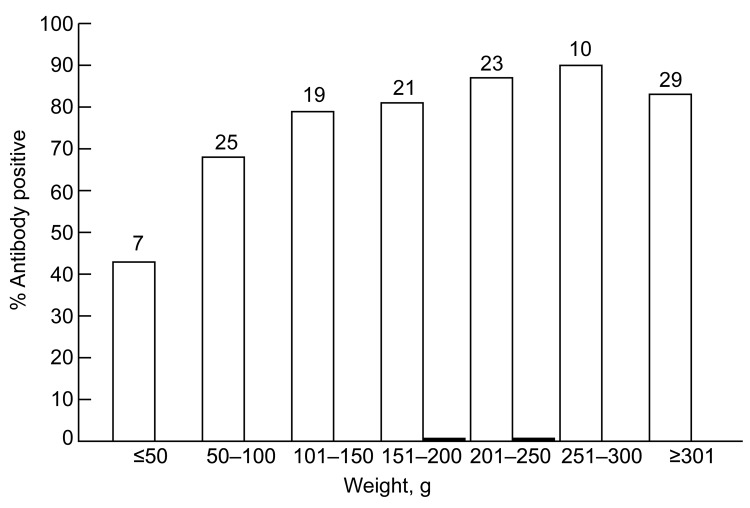
Relationship between prevalence of antibodies against hepatitis E virus (HEV) and weight of *Rattus norvegicus* rats trapped in Los Angeles, California, USA. Rats reach sexual maturity at a weight of ≈150–200 g. White bars indicate IgG, and black bars indicate IgM. Numbers at the top of each bar indicate sample size.

We had reported that wild rats trapped in Baltimore, Maryland, and the Hawaiian Islands had prevalences of antibodies against HEV of 77%–94% (*12*). We tested 134 serum samples from *R. norvegicus* rats trapped in urban Los Angeles. Donor rats were a mixture of male and female animals and adults and juveniles weighing 26–508 g. Of these animals, 105 (78.4%) were positive for IgG against HEV (with or without IgM against HEV), 2 (1.5%) were positive for IgM against HEV only, and 27 (20.1%) were seronegative when tested by ELISA with antigen derived from human HEV (*14*). As we described (*12*), prevalence of antibodies against HEV increased with weight as a measure of age, and ≈50% of the youngest rats were already positive for antibodies against HEV (Figure 1).

Because HEV is neutralized by antibodies against HEV, seronegative serum samples or IgM-positive serum from animals early after infection offer the greatest chance of recovering infectious virus. Therefore, 6 pools of serum were prepared from 27 seronegative rats, and 250 μL of serum from each pool and individual serum samples from 2 IgM HEV-positive rats and 5 IgG/IgM–positive rats weres used to infect Sprague-Dawley rats. All 13 rats remained negative for HEV RNA, and only 3 rats (peak ELISA titers 100–400) seroconverted. Two of the seroconverted rats had received pooled seronegative serum, and the other had received IgM-positive serum.

### Passage of HEV to Rats

Feces obtained during the first 4 weeks postinfection from the 3 seroconverting rats (B76, B79, and B84) were homogenized and pooled, and 200 µL was used to inject 4 additional rats each. Only 3 of 12 rats injected with feces from rats B76 or B84 seroconverted. Serum was less efficient at transmitting virus, and 0 of 8 rats were infected. To determine the duration of shedding, infectious virus was identified by seroconversion of rats that had been infected intravenously with 200 µL of 10% suspensions of individual serial fecal samples obtained every other day from rats B76 and B84 during the first 4 weeks postinfection; fecal suspensions were also tested by RT-PCR. Feces from rat B76 were positive for >11 days and feces from rat B84 were positive for >9 days (Table 2). Periods of PCR positivity coincided with intervals of transmissibility on the basis of seroconversions in recipient rats. However, none of the recipient rats became viremic.

**Table 2:** Rat HEV in serial fecal samples of experimentally infected laboratory rats, Los Angeles, California. USA*

Animal	Day postinfection														
	7	9	11	13	15	17	19	21	23	25	27	29	31	33	35
Rat B76															
Infectious†	NT	NT	NT	–	+	–	+	–	–	–	–	–	–	NT	NT
RT-PCR	NT	NT	NT	+	+	+	+	+	+	–	–	–	–	NT	NT
Serum antibody against HEV	NT	NT	NT	–	–	–	–	–	–	–	–	+	+	+	+
Rat B84															
Infectious†	+	–	+	+	+	–	–	–	–	–	NT	NT	NT	NT	NT
RT-PCR	+	+	+	+	+	–	–	–	–	–	NT	NT	NT	NT	NT
Serum antibody against HEV	–	–	–	–	–	–	–	+	+	+	+	+	+	+	+

*HEV, hepatitis E virus; NT, not tested; –, negative; +, positive; RT-PCR, reverse transcription PCR. †As measured by transmission to another rat.

Because serum and feces were poor sources of transmissible virus, we tested other clinical materials. Groups of rats injected with fecal pools from rat B76 and rat B84 were exsanguinated on various days, and serum, liver, and intestinal contents were harvested. Serum from these rats was injected into individual rats, which were tested for seroconversion. Only 2 serum samples (from rats B300 and B182) transmitted virus to a new rat. The liver of rat B182 was used for further transmission studies.

To establish a more robust infection, we injected nude rats, which lack a functional adaptive immune system. Nineteen nude rats were injected with 200 µL of a 10% liver homogenate from rat B182 at a dilution of 10^−1^ and 1 rat was killed daily (days 2–20). We then used 200 µL of a 10^−2^ dilution of serum from the killed rats to infect Sprague-Dawley rats. Only 3 of these rats seroconverted, indicating that only 3 of the nude rats (killed on days 13, 15, and 19) had infectivity titers >10^2^. One of these 3 nude rats, rat B350, was further studied.

### Titer of Rat HEV

To determine the infectivity titer of rat HEV in liver, serum, and feces of selected infected rats, reverse titrations were performed with Sprague-Dawley rats and were monitored for seroconversion (Table 3). In Sprague-Dawley rats, 50% rat infectivity doses (RID_50_) of 10^4^–10^5^/g of liver tissue were observed; in nude rat B350, a titer >10^6.0^/g of liver and a titer of 10^3.7^ in serum were detected. Titers of virus in feces and intestinal contents of Sprague-Dawley rats were <10^1^ and <10^3^, respectively. Feces from nude rats were not tested. These samples were also titered for PCR positivity by qRT-PCR (Table 3). PCR titers of rat HEV paralleled infectivity titers but averaged an ≈10–100-fold higher titer.

**Table 3:** Titers for HEV in samples from laboratory rats, Los Angeles, California, USA*

Sample type and source	log_10_ ID_50_	log_10_ RT-PCR_50_
Feces		
Rat B76†	<1	3.4
Rat B84†	<1	3.4
Intestinal contents: Rat B182		
Small intestine	<3	4.9
Cecum	<3	5.4
Colon	<3	4.9
Serum		
Rat B182	ND	3.7
Rat B300	ND	<2.2
Rat B350	3.7	4.7
Liver		
Rat B182	4.7	7.2
Rat B300	4.2	5.7
Rat B350	6.2	7.7

*Values are per milliliter or per gram. HEV, hepatitis E virus; ID_50_, 50% infectious dose; RT-PCR_50_, 50% reverse transcription PCR titer; ND, not determined. †Serum from wild rats was injected into laboratory rats B76 and B84. Samples from these 2 rats were serially passaged into other laboratory rats (wild rat → rat B76 → rat B300; wild rat → rat B84 → rat B182 → rat B350).

### Sequence of Rat HEV

A 327-nt product was amplified from the liver of rat B350 by nested RT-PCR and directly sequenced to yield the consensus sequence. The B350 rat virus sequence was as genetically similar to the 2 rat sequences from Germany as they were to each other at the nucleotide and amino acid levels (Table 4).

**Table 4:** Pairwise identity comparisons of a 327-nt fragment amplified from ORF1 of rat HEV, Los Angeles, California, USA*

Strain	% Identity		
	Rat B350	Ger 715	Ger 719
Rat B350		87.5	85.3
Ger 715	96.3		86.2
Ger 719	96.3	95.4	

*Values above the diagonal are nucleotide identities; values below the diagonal are amino acid identities. ORF, open reading frame; HEV, hepatitis E virus. Rat B350, GenBank accession no. JF516246; Ger 715, accession no. GQ504009.1; Ger 719, accession no GQ504010.1.

### Effect of Infection on Liver Enzyme Levels

**Figure 2:**
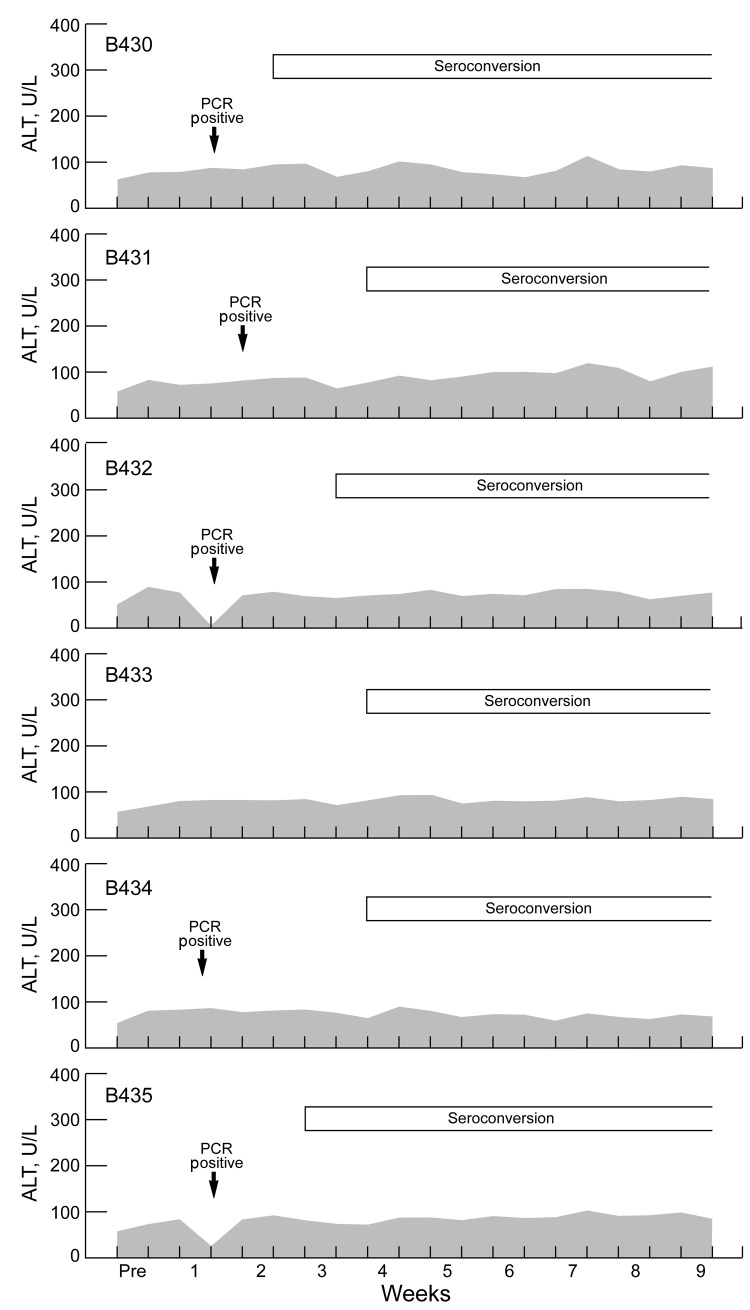
Correlation between virus infection and serum levels of alanine aminotransferase (ALT) (shaded areas) in rats infected with hepatitis E virus, Los Angeles, California, USA. Six Sprague-Dawley rats (B430–5) were infected with a 10^4.5^ 50% rat infectious dose of rat HEV and tested 2×/wk for evidence of infection and hepatitis. PCR results were positive for only half a week in 5 of the 6 rats. Pre, preinfection.

We have shown that some mammalian HEV strains show a dose response: higher doses (>10^4^ infecting virus) are more likely to be associated with higher serum liver enzyme levels. To determine whether this phenomenon was true also for rat HEV, we infected 6 Sprague-Dawley rats with 200 µL of liver homogenate from rat B350 that contained 10^4.5^ RID_50_ of rat HEV. Animals were bled 2×/wk, and levels of alanine aminotransferase, γ-glutamyl transpeptidase, and isocitrate dehydrogenase were measured for 3 months. All 6 animals seroconverted 2.0–3.5 weeks (mean 3.0 weeks) postinfection (Figure 2). As reported, liver enzyme levels varied considerably, but seroconversion and liver enzyme levels were not temporally associated. Thus, these infections were biochemically inapparent infections.

### Histologic Evaluation

**Figure 3:**
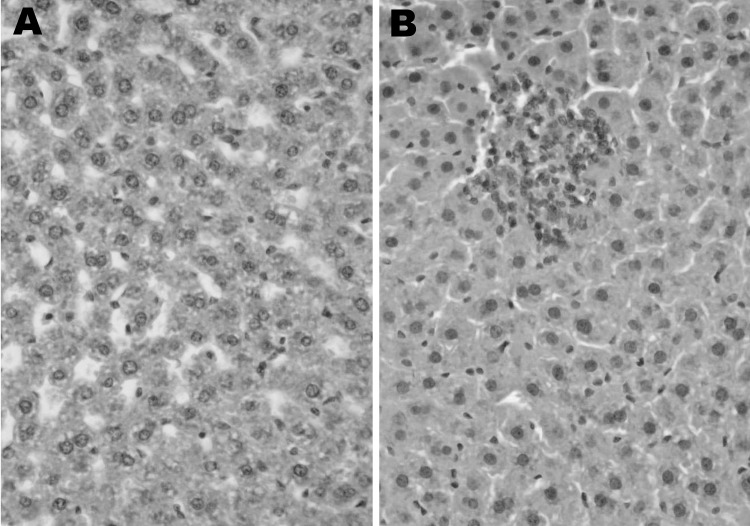
Histologic analysis of infection with rat hepatitis E virus, Los Angeles, California, USA. Hematoxylin and eosin–stained sections of liver from a healthy rat (A) and a rat acutely infected with rat HEV (B). Original magnification ×200.

Two HEV-infected rats (B182 and B300) and 2 uninfected Sprague-Dawley rats were examined under code for histologic evidence of hepatitis. The 2 uninfected rats had essentially normal livers. Rat B182 had parenchymal foci of necrosis and aggregates of lymphocytes and Kupffer cells in hepatic lobules and had mild portal inflammation (Figure 3). Rat B300 had similar but less obvious lesions. This mild hepatitis was consistent with normal liver enzyme levels measured in serum of these animals.

### Transmission of Rat HEV to Nonhuman Primates

Because rats and humans often share the environment, especially in inner cities, we tested whether rat HEV was transmissible to nonhuman primates. Seronegative rhesus monkeys, which are surrogates of humans, were injected intravenously with rat liver homogenate containing 10^3.5^ RID_50_ of rat HEV from rat B182 or with 10^5.2^ RID_50_ of rat HEV from rat B350. The animals were monitored for 15 weeks for seroconversion by ELISA and for genomic RNA by qRT-PCR. The animals remained negative (Table 5). Thus, rat HEV does not appear to be transmissible to rhesus monkeys.

**Table 5:** Results of testing for transmission of rat HEV to rhesus monkeys, Los Angeles, California, USA*

Inoculum	ID_50_†	No. injected	No. with HEV RNA or antibodies against HEV
Rat B182	10^3.5^	2	0
Rat B350	10^5.2^	2	0

*HEV, hepatitis E virus. The 50% infectious dose (ID_50_) was administered intravenously. †In liver homogenate and titered in rats.

## Discussion

Previous studies of HEV in rats have been fraught with controversy. The earliest report linked serologic evidence of HEV in rats near a village in the former Soviet Union with an epidemic of hepatitis E in the village (*20*). Later studies reported transmission of HEV in human feces from Nepal (presumably genotype 1) to laboratory rats (*21*) and isolation of genotype 1 HEV sequences from rats trapped in Nepal (*22*). However, the second study was retracted (*23*).

To determine whether rats were susceptible to recognized mammalian strains of HEV, we intravenously injected laboratory rats with human genotype 1 strains of HEV from Sargodha, Pakistan (*15*); Akluj, India (*17*); and Kashi, People’s Republic of China (*16*); a human genotype 2 strain from Mexico (*19*); and a swine genotype 3 strain from Illinois, USA (*18*). Infectious titer of virus administered ranged from ≈10^2^ to 10^5^. None of the animals had evidence of infection, which suggested that rats are not readily susceptible to infection with other mammalian HEVs.

Nevertheless, as reported recently, rats can be infected by HEV strains (*11*). Using published primers, we amplified HEV genomic sequence from 1 of 2 HEV strains isolated in urban Los Angeles. This sequence was similar to sequences isolated from 2 rats in Hamburg, Germany; the virus sequence from Los Angeles was as similar to the 2 sequences from Germany as they were to each other. All 3 strains had only ≈60% identity with other mammalian strains, which suggested that rat HEV comprises a new HEV genotype.

On the basis of our extensive attempts to identify the virus in naturally infected wild caught and experimentally infected laboratory rats, we concluded that rat HEV infections were not robust and that the magnitude and duration of viremia and fecal shedding were less than that usually observed in infections with the other mammalian HEV genotypes. A low titer of rat HEV in rat feces in Germany was also reported (*11*). Rat HEV caused minimal hepatitis in experimentally infected animals; liver enzyme levels seldom increased above baseline levels, and histopathologic lesions during acute infections, although present, were minimal and not associated with clinical disease. Nevertheless, age-specific antibody prevalence in rats suggests that they are easily infected in their natural environment, and most are infected as juveniles and young adults in a pattern similar to that seen for acquisition of antibody against HEV in swine and humans in hepatitis-endemic areas (*24**,**25*).

Antibody against HEV in rats was usually directed against epitopes other than the major neutralization epitope in the carboxy portion of a genotype 1 capsid protein (S.U. Emerson, unpub. data). Seroconversion was relatively sensitive in identifying HEV infection in rats; it was in some cases more sensitive than detecting viremia by PCR. However, PCR was ≈10–100-fold more sensitive than infectivity titrations for quantifying HEV, a difference that is common for many virus infections. Overall, PCR confirmed that the magnitude and duration of viremia and viral shedding are not robust in rats. Whether capsid antigen expressed by rat virus will result in a more specific and sensitive assay for rat HEV antibody and whether it will help to better define the specificity of existing tests for antibodies against HEV in humans should be determined.

The high prevalence of antibodies against HEV in humans living in countries to which HEV is not endemic suggests that HEV infection in such areas might be zoonotic. Nevertheless, a direct association between HEV infection in animals and hepatitis E in humans has been limited, for the most part, to exposure to swine through eating undercooked pork and especially undercooked offal or through environmental exposure to swine feces. However, most persons do not eat undercooked pork or come in contact with swine, and their exposure, especially among those living in inner cities or in cultures without pigs, remains an enigma. In such settings, exposure to rats could be the missing link to HEV infection.

To determine whether this link exists, we attempted to transmit rat HEV to rhesus monkeys, a surrogate of humans that are highly susceptible to mammalian genotypes 1–4, including swine HEVs (*26**–**28*). Although we administered >100,000 infectious doses of virus intravenously to monkeys, they were not infected, as shown by lack of viremia and failure to develop antibodies against HEV. We also demonstrated similar lack of transmissibility of avian HEV to rhesus monkeys in previous collaborative studies (*29*), and we believe that these studies suggest a lack of zoonotic threat to humans from either avian or rat HEV.
